# Mental wellbeing among urban young adults in a developing country: A Latent Profile Analysis

**DOI:** 10.3389/fpsyg.2022.834957

**Published:** 2022-09-02

**Authors:** Thao Thi Phuong Nguyen, Tham Thi Nguyen, Vu Trong Anh Dam, Thuc Thi Minh Vu, Hoa Thi Do, Giang Thu Vu, Anh Quynh Tran, Carl A. Latkin, Brian J. Hall, Roger C. M. Ho, Cyrus S. H. Ho

**Affiliations:** ^1^Institute for Global Health Innovations, Duy Tan University, Da Nang, Vietnam; ^2^Faculty of Pharmacy, Duy Tan University, Da Nang, Vietnam; ^3^Institute of Health Economics and Technology, Hanoi, Vietnam; ^4^Center of Excellence in Evidence-Based Medicine, Nguyen Tat Thanh University, Ho Chi Minh City, Vietnam; ^5^Institute for Preventive Medicine and Public Health, Hanoi Medical University, Hanoi, Vietnam; ^6^Bloomberg School of Public Health, Johns Hopkins University, Baltimore, MD, United States; ^7^Global and Community Mental Health Research Group, New York University (Shanghai), Shanghai, China; ^8^Institute for Health Innovation and Technology (iHealthtech), National University of Singapore, Singapore, Singapore; ^9^Department of Psychological Medicine, Yong Loo Lin School of Medicine, National University of Singapore, Singapore, Singapore

**Keywords:** mental health, urban population, adults, Latent Profile Analysis, Vietnam

## Abstract

**Introduction:**

This study aimed to explore the mental wellbeing profiles and their related factors among urban young adults in Vietnam.

**Methods:**

A cross-sectional study was conducted in Hanoi, which is the capital of Vietnam. There were 356 Vietnamese who completed the Mental Health Inventory-5 (MHI-5) questionnaire. The Latent Profile Analysis (LPA) was used to identify the subgroups of mental wellbeing through five items of the MHI-5 scale as the continuous variable. Multinomial logistic regression was used to determine factors related to subgroups.

**Results:**

Three classes represented three levels of MHI-5 score, which included “Poor mental health,” “Fair mental health,” and “Good mental health,” were, respectively, 14.3, 46.6, and 39.0%. Compared to a low household economy, participants with an average household economy had 2.11 and 4.79 times higher odds of being in a good mental health class relative to fair and poor mental health classes. Respondents with more than two acute symptoms had 3.85 times higher odds of being in a good mental health class relative to a poor mental health class, as compared to those without acute symptoms. Regarding the measurement of the Perceived Social Support Scale (MSPSS), people having support from their family had 1.80 and 2.23 times higher odds of being in classes of fair and good mental health relative to the poor mental health class; and participants having friend support also had 1.87 times higher odds of being in a good mental health class compared with the fair mental health class, as the MSPSS score increased by 1 unit. People with Rosenberg’s self-esteem scale increased by 1 score, those who had 1.17, 1.26, and 1.47 times higher odds of being in a good compared to fair mental health class, fair compared to poor mental health class, and good compared to poor mental health class, respectively.

**Conclusion:**

Our findings were given to promote a new classification method for mental health screening among the general population. The current findings could be used as evidence to develop policies and plans that focus on encouraging early screening for mental health problems among the general young population in the future.

## Introduction

The World Health Organization (WHO) defines mental health as “a state of wellbeing in which the individual realizes his or her own abilities, can cope with the normal stresses of life, can work productively, and is able to make a contribution to his or her community” (World Health Organization, 2010). Mental health issues affect not only a small segment of the population but also society as a whole. Therefore, ensuring the mental health of all residents is a major challenge for global development and health care systems across nations (World Health Organization, 2003). In Northern Vietnam, a longitudinal study from 2006 to 2013 indicated a depressive trajectory among adolescents and young adults (Bui et al., 2018). Furthermore, the prevalence of suicidal thoughts and having suicide plans were, respectively, 14.1 and 5.7%, which was quite high in this vulnerable population (Le et al., 2016). According to a review by UNICEF, the prevalence of mental health issues among young Vietnamese people ranged from 8 to 29% (UNICEF, 2018). Numerous national surveys among Vietnamese youth were conducted in nearly all regions between 2003 and 2010 (Niemi et al., 2010; Weiss et al., 2012; Thai et al., 2020). However, most of the mentioned studies just described the prevalence of mental health problems among young people without any information on the general population’s classification or screening of mental health issues. Moreover, these studies have not yet provided in-depth analytical methods to identify the related factors affecting the mental health of young Vietnamese people.

Nowadays, various screening tools have been developed to assist in detecting psychological and psychiatric problems. These tools were mainly based on the principles of symptom scoring and had good sensitivity to detect signs of poor mental health. Some of these could be completed in only a few minutes with 20 to 30 items, even shorter instruments, but showed good performance to detect mental health problems (Berwick et al., 1991; Whooley et al., 1997; Rumpf et al., 2001). The Mental Health Inventory-5 (MHI-5) as a five-item questionnaire is used to assess general community mental health (Berwick et al., 1991). This short instrument was developed from both versions of the Medical Outcome Study (MOS) questionnaires: MOS Short Form 20 (Stewart et al., 1988) and MOS Short Form 36 (Ware and Sherbourne, 1992). In a prior study, MHI-5 had been demonstrated to be as good as the more comprehensive and commonly used general health questionnaire (GHQ) (Goldberg and Blackwell, 1970). The performance of MHI-5 was assessed as similar to GHQ-12 in a previous study by McCabe et al. (1996). However, they did not find which might be used as the gold standard for psychiatric screening (Goldberg, 1988). The MHI-5 scale has been developed and validated in the Vietnamese version through an SF-36 survey of 1610 Vietnamese aged over 15 years (Watkins et al., 2000). Furthermore, this research just focuses on testing the psychometric properties of a culturally relevant translation of the medical outcomes. Similarly, this scale has also been used for screening mental health problems among women (Collier et al., 2020), the elderly (Nguyen and Kruse, 2012), or cardiovascular patients (Van Nguyen et al., 2020) in Vietnam.

In a large cohort of the surveyed population, it was likely that different profiles of mental levels existed, which means individuals were classified into the same mental health classes. A previous study regarding mental screening among American adolescents showed three latent classes included flourishing mentally healthy, moderately mentally status, and languishing status (Keyes, 2006). Those who were flourishing mentally group had the greatest psychosocial functions, whereas those who were languishing had the most depressive symptoms and behavioral issues (Keyes, 2006). The majority of mental health screening studies use MHI-5 based on cut-off points (Berwick et al., 1991; Rumpf et al., 2001; Cuijpers et al., 2009) or exploratory factor analysis (EFA) (Rivera-Riquelme et al., 2019) to test the mental health factor structures of participants. However, these studies had not indicated latent profiles of mental levels which might exist. Latent Profile Analysis (LPA) has the potential for latent variable modeling as well as creating and expanding theoretical thinking on the existence of various profiles in variables (Hirschi and Valero, 2015; Gillet et al., 2018). To the best of our knowledge, there are very few studies that specifically target LPA on the MHI-5 scale. After reviewing the literature, we found two studies relative to this topic, which included a longitudinal study of 9,683 women in 2015 (Leigh et al., 2016) and a cross-sectional study of 409 young Finnish men in 2022 (Appelqvist-Schmidlechner et al., 2022). Therefore, this study aimed to explore the mental wellbeing profiles and their related factors among urban young adults in Vietnam.

## Materials and methods

### Study setting and participants

The study was designed as a cross-sectional investigation of the young Vietnamese population from May 2020 to September 2020. A research facility was established at the Institute for Preventive Medicine and Public Health, Hanoi Medical University, which was implemented to collect voluntary participants.

Participants were recruited following the inclusion criteria: (1) aged 18 to 25; (2) currently living in Hanoi, Vietnam; and (3) willing to sign informed consent and engage in the research. Exclusion criteria included: (1) participants who got serious illnesses; and (2) could not answer questions. The study used both methods of random and convenience sampling, with a completion rate of approximately 100%. During the data collection period, once participants agreed to participate in this study by signing informed consent, a face-to-face interview was conducted for 20–25 min by investigators who were well-trained to use questionnaires. Additionally, the interview took place in a closed room, to ensure privacy and limit outside influences.

We used the registrar’s database of all currently enrolled students at Hanoi Medical University in 2020, with approximately 6,000 students and 1,000 graduate or professional students. A preliminary list was established based on the simple random sample selection, with 10% of students and 20% of graduate/professional students aged 18 to 25 living in Hanoi, Vietnam. Therefore, 600 students and 200 graduate or professional students were collected for the preliminary list. There were 80% of students and 90% of graduate/professional students who had phone numbers. A phone message about the study and a participating invitation would be randomly sent to people on the preliminary list. Approximately 20.8% of students and 55.5% of graduate/professional students responded that they could participate in our study. Those were invited to the research facility and conducted a face-to-face interview. Before starting the interview, participants would be given a written consent form to sign if they decided to participate. There were three research assistants who conducted the direct interviews. As a consequence, the study sample size was collected from a preliminary list of 200 participants, including 100 undergraduates and 100 graduate/professional students. To expand and diversify the study sample, after finishing the interview, we requested participants invite others, such as friends or relatives, to participate in the survey. As a result, the present study recruited a further 156 people from the initial group. A total of 356 participants were recruited for our study.

### Measurement

To collect data, a semi-structured questionnaire consisting of seven main components was developed, including (1) socio-demographic characteristics; (2) alcohol consumption; (3) health conditions; (4) General Self-efficacy scale; (5) Multidimensional Scale of Perceived Social Support scale; (6) Rosenberg Self-Esteem scale; and (7) Mental Health Inventory scale. Before collecting data, this questionnaire was piloted on 10 people of different genders, ages, and occupations to secure the text and logical issue of each question and modify the question with unclear meaning.

#### Socio-demographic

Respondents answered questions including gender (male/female), age (unit; year), education level that participants completed (high school or lower/tertiary and upper), marital status (single/married), job (students/others), and household economy (low/average/high).

#### Alcohol consumption

We used the Alcohol Use Disorders Identification Test-Consumption (AUDIT-C) scale to evaluate the alcohol consumption of participants. The AUDIT-C scale has been used in many studies in Vietnam. Moreover, the Vietnamese version of this tool was also validated elsewhere (Giang et al., 2005, 2008; Tran et al., 2013, 2014). This tool includes three questions with an overall score of 12 points. People are considered Hazardous drinking when the total score of questions 1 and 2 accounts for four points (male) or three points (female). The indicator of Binge drinking is determined when respondents answer the frequency of consuming six or more drinks at one time (Bush et al., 1998). The Cronbach’s alpha of the AUDIT-C scale was 0.71 in the current study.

#### Health conditions

To explore information about acute symptoms and chronic diseases that participants experienced, two questions were developed, including (1) “Have you ever been experienced any acute symptoms during the last 4 weeks?” (Yes/No), and (2) “Have you ever been diagnosed with any chronic diseases during the last three months?” (Yes/No). If participants indicated “yes” to the above question, they were then asked to self-report their symptoms or conditions. These acute symptoms could include headaches, backaches, allergies, constipation, coughs/sore throats, sneezes/runny noses, fever, helminth infections, diarrhea, gynecologic diseases, skin diseases, eye diseases, etc., that they experienced in the last 4 weeks and chronic diseases they experienced in the last 3 months. The chronic diseases consist of hypertension, cardiovascular disease, diabetes, cancer, asthma, epilepsy/psychiatry, stomach/digestive, chronic obstructive pulmonary disease, etc.

#### General Self-Efficacy scale (GSE)

A 10 items of the GSE are created to assess a general sense of perceived self-efficacy with the aim in mind to predict coping with daily hassles as well as adaptation after experiencing all kinds of stressful life events. For each item, a four-point Likert scale is used, ranging from 1 (not at all true) to 4 (exactly true). The total score of GES is calculated by summing all items. After calculating, the score ranged from 10 to 40, with a higher score indicating more self-efficacy (Schwarzer and Jerusalem, 1995). Furthermore, the internal reliability of GSE was reported to be between 0.76 and 0.90. The GSE has been used and validated as a unidimensional construct in some countries. This tool was considered an easy-to-manage and comprehensive evaluation measurement of self-esteem among young adults (Luszczynska et al., 2005; Leung and Leung, 2011; Teo and Kam, 2014). In Vietnam, this tool had also been utilized in previous studies (Goto et al., 2010). In this study, the Cronbach’s alpha for this scale was good at 0.84.

#### Multidimensional Scale of Perceived Social Support (MSPSS) scale

It is a self-reported measure of subjectively assessed social support. The MSPSS consists of 12 items that are divided into three subdomains [including friends (4 items), family (4 items), and significant other (4 items)]. Each item is measured by 7 Likert points from 1 (very strongly disagree) to 7 (very strongly agree) (Zimet et al., 1990). The total score of each domain is summed and then divided by 4 (Zimet, 2016). The higher score of each domain indicates the higher perceived social support. To date, MSPSS has been widely used and validated in some countries, including Vietnam (Wongpakaran et al., 2011; Guan et al., 2014; Lam et al., 2021; Cartwright et al., 2022; Nguyen et al., 2022). In this study, the Cronbach’s alpha for this scale was good at 0.91.

#### Rosenberg Self-Esteem scale

A 10-item scale that measures global self-worth by measuring both positive and negative feelings about oneself. Items 2, 5, 6, 8, and 9 are used to measure negative feelings, while other items are utilized to assess positive feelings about A 10-item scale that measures global self-worth by measuring both positive and negative feelings about oneself. For negative items, a four-point Likert scale is used, including 1-strongly agree, 2-agree, 3-disagree, and 4-strongly disagree. Meanwhile, the options for positive items are reversed, ranging from 1-Strongly Disagree, 2-disagree, 3-agree, and 4-strongly agree. Finally, the total score of all items is calculated, with a higher score indicating higher self-esteem (Kielkiewicz et al., 2020). The Rosenberg self-esteem scale has been validated in some countries, including Vietnam (Martín-Albo et al., 2007; Wongpakaran and Wongpakaran, 2012; Mullen et al., 2013; Kourakou et al., 2021). This tool has also been widely applied in some studies (Nguyen et al., 2010; Nguyen D. T. et al., 2019). In this study, the Cronbach’s alpha for this scale was 0.70.

#### Mental Health Inventory-5 (MHI-5)

MHI-5 is considered a simple and valid instrument to detect depressive symptoms among the general population (Yamazaki et al., 2005; Kelly et al., 2008; Cuijpers et al., 2009). This scale consists of 5 dimensions, which is known as a subscale of the Short-Form Health Survey Questionnaire (SF-36) (Ware and Sherbourne, 1992; Stoll et al., 2001). In particular, the MHI-5 investigates the mental health status during the last month since the interview, with items including (1) feeling nervous; (2) loss of pleasure; (3) feeling calm and peaceful; (4) feeling downhearted and blue; and (5) feeling happy. For each item, a 6-point Likert scale is used, including 1 “all of the time,” 2 “most of the time,” 3 “a good bit of the time,” 4 “some of the time,” 5 “a litter of the time,” and 6 “none of the time” (Berwick et al., 1991). However, the scores of items (3) and (5) talking about positive feelings are reversed, with a range of scores from 1 “none of the time” to 6 “all of the time.” In studies with the Vietnamese population, the MHI-5 scale was also applied in some studies to assess mental health problems (Daly et al., 2019). In this study, the Cronbach’s alpha of MHI-5 was good at 0.84.

### Data analysis

Latent Profile Analysis is a type of categorical latent variable modeling that focuses on identifying latent subpopulations within a population based on a collection of factors (Collins and Lanza, 2009; Wang and Hanges, 2011; Howard and Hoffman, 2018). LPA has the potential to answer specific research issues as well as to create and expand theoretical thinking on the existence of various profiles in variables (Hirschi and Valero, 2015; Gillet et al., 2018). In particular, LPA assumes that each person may be classified into subpopulations with varying degrees of probability based on the information collected from participants. By using LPA, categorical latent variables are identified, so investigators can get a parsimonious representation of structures in the form of groups (Woo et al., 2018). The MHI-5 scale is a comprehensive assessment instrument for screening the mental wellbeing in the community (Daly et al., 2019), but there is a lack of investigations that could distinguish subgroups on this measurement. Therefore, in this study, LPA was applied to determine group of people (categorical latent variables) who had similar responses to five items of the MHI-5 scale.

Latent Profile Analysis was used to identify the subgroups of mental wellbeing status, with MPLUS version 8.5 (Asparouhov and Muthén, 2021). Five items of the MHI-5 scale as a continuous variable were selected to perform LPA (Rhemtulla et al., 2012). A model containing one to six profiles was used to determine the number of suitable classes. To determine the number of latent profiles with the best accurate classification and goodness of fit, each model was assessed by the following several indices: the lower Akaike Information Criterion (AIC) (Akaike, 1987), the lower Bayesian Information Criterion (BIC) (Schwarz, 1978), the lower sample-size adjusted Bayesian information criterion (aBIC) (Sclove, 1987), the higher entropy value (Celeux and Soromenho, 1996), the significant bootstrapped likelihood ratio test (BLRT) (Nylund et al., 2007), the significant Lo– Mendell–Rubin adjusted likelihood ratio test (LMR-LRT), the significant Adjusted Lo-Mendell-and Rubin likelihood ratio test (LMRa-LRT) (Lo et al., 2001). These tests were calculated for model fit decision purposes by comparing models where a significant *p*-value (*p* < 0.05) showed that a k-class model fitted better than the (k-1) – class model (Toker, 2016).

Both descriptive and analytical statistics were used to address the main aims of the study by STATA version 16. Continuous variables were presented as mean and standard deviation (SD), while categorical variables were presented as frequency with percentage. Descriptive, analytical statistics and multinomial logistic regression between all variables were calculated using STATA version 16 (StataCorp LP, College Station, TX, United States). We used Kruskal-Wallis tests for continuous variables and χ^2^ tests for categorical variables to compare the differences between three classes of mental wellbeing and some characteristics. Multinomial logistic regression models were utilized to determine factors related to the level of mental wellbeing among participants. The social-demographic characteristics, health status, alcohol consumption, General Self-efficacy cale, Multidimensional Scale of Perceived Social Support, and Rosenberg self-esteem scale were all potential covariates for the full model. A *p* value (*p*) < 0.05 was considered statistically significant.

### Ethical consideration

This study was ethically approved by the Institutional Ethical Review Board at Hanoi Medical University on March 13, 2020. Informed consent would be supplied to participants before starting the study. People would sign the written consent if they decided to participate. Participation was completely voluntary; they could feel free to decline to join for any reason. Even after signing the consent form, they could also stop participating in this project at any time or refuse to answer any individual questions. Collected data was saved in a secured system and served for study purposes only.

## Results

A study sample consisting of 356 participants with a mean age of 22.03 (SD = ± 3.21) was assessed for mental health status by using the MHI-5.

### The characteristics of the MHI-5 scale

Table 1 describes the percentage of responses and the average score of each question according to the 6 answer options of the MHI-5 scale. In terms of questions 1, 2, and 4, the majority of participants answer with the levels of “some of the time” and “a little of the time.” By contrast with questions 3 and 5, most respondents reported “most of the time” and “a good bit of time.” On the MHI-5 scale, the mean score for each item ranged from 3.98 to 4.75 points.

**TABLE 1:** The characteristics of the MHI-5 scale.

	Percentage of each answer option (%)						Mean	SD
	
	All of the time	Most of the time	A good bit of time	Some of the time	A little of the time	None of the time		
ITEM 1. Feeling nervous	0.84	1.97	19.10	30.34	32.87	14.89	4.37	1.07
ITEM 2: Loss of pleasure	0.28	0.84	6.46	32.58	34.83	25.00	4.75	0.94
ITEM 4. Feeling downhearted and blue	0.28	0.28	9.83	25.84	44.66	19.10	4.72	0.91

	**None of the time**	**A little of the time**	**Some of the time**	**A good bit of time**	**Most of the time**	**All of the time**	**Mean**	**SD**

ITEM 3. Feeling calm and peaceful	1.12	10.11	16.85	37.92	29.78	4.21	3.98	1.07
ITEM 5. Feeling happy	0.84	8.15	14.89	38.76	29.49	7.87	4.20	1.07

### Comparison of Latent Profile Analysis models with different latent classes based on model selection statistics and the most suitable model

Table 2 demonstrated a latent profile class with the largest model of four classes of the MHI-5 scale. Several models were selected according to the suitable indicators (AIC, BIC, and aBIC) and test values (LMR-LPT, LMRa-LRT, and BLRT). A model with three classes was the most suitable based on the values of fit indexes and tests. In particular, the indicators of AIC, BIC, and aBIC reduced significantly when more classes were added to the model. Therefore, the model with two and three classes was preferred. Although the four class-models showed the favored values of AIC, BIC, aBIC, examining three tests (LMR-LPT, LMRa-LRT, and BLRT) determined three class-models with *p* < 0.05, which was the most appropriate model.

**TABLE 2:** Comparison of LPA models with different latent classes based on model selection statistics and the most suitable model.

Number latent class	AIC	BIC	aBIC	Entropy	LMR-LRT (*p* value)	LMRa-LRT (*p*- value)	BLRT (*p*- value)
1- Class	5106.92	5145.67	5113.94	–	–	–	–
2- Class	4611.02	4673.02	4622.26	0.82	<0.01	<0.01	<0.01
3-Class ***	**4495.26**	**4580.51**	**4510.72**	0.78	**0.01**	**0.01**	**<0.01**
4-Class	4442.06	4550.56	4461.73	0.81	0.13	0.14	<0.01

The *** mean suitable result has been selected. The bold value means a suitable result has been selected.

### Three factors of the MHI-5 scale were obtained from Latent Profile analysis

**FIGURE 1:**
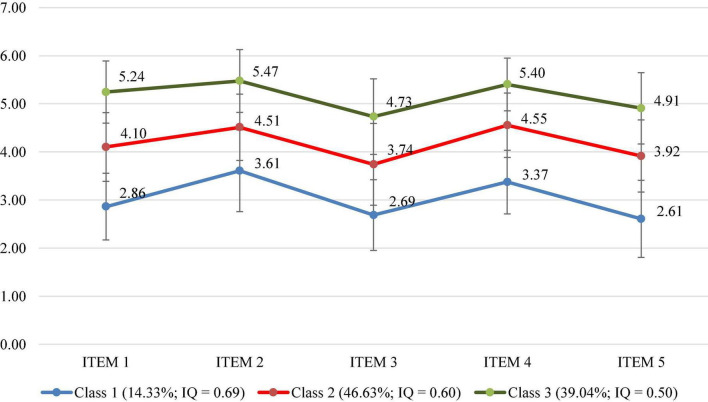
LPA on the three classes of the MHI-5. Class 1, Poor mental health; Class 2, Fair mental health; Class 3, Good mental health; IQ, Inter-quartile range; ITEM 1, Feeling nervous; ITEM 2, Loss of pleasure; ITEM 3, Feeling calm and peaceful; ITEM 4, Feeling downhearted and blue; and ITEM 5, Feeling happy.

In Figure 1, the total mean score MHI-5 from five questions was divided into three classes, which included “poor mental health,” “fair mental health,” and “good mental health.” The respective mean scores of the three classes were 14.33% (poor mental health), 46.63% (fair mental health), and 39.04% (good mental health). In terms of poor mental health class, the mean score of items 1, 2, 3, 4, and 5 was reported at 2.86 ± 0.69, 3.61 ± 0.85, 2.68 ± 0.73, 3.37 ± 0.66, and 2.61 ± 0.80, respectively. Regarding the fair mental health class, the mean score of item 1 was 4.10 ± 0.73, item 2 was 4.51 ± 0.68, item 3 was 3.74 ± 0.85, item 4 was 4.55 ± 0.66, and item 5 was 3.92 ± 0.75. Compared to other groups, each item of MHI-5 scale in the good mental health class had the higher score, with 5.24 ± 0.65 (item 1), 5.47 ± 0.65 (item 2), 4.73 ± 0.79 (item 3), 5.40 ± 0.55 (item 4), and 4.91 ± 0.73 (item 5).

### Characteristics of participants according to three classes from the MHI-5 scale

Table 3 describes some characteristics among 356 participants regarding the MHI-5 scale. The majority of respondents were female (72.08%), students (89.61%), and educated at the level of high school or lower (83.15%). The majority of participants with more than two acute symptoms occupied 60.11%. Regarding the AUDIT-C scale, Hazardous and Binger drinking indicators were found to be 77.12 and 84.27%, respectively. In terms of the MSPSS scale, participants who belong to the good mental health class have higher mean points (of all three domains, including family, friends, and significant others) than the two remaining classes, with mean values of 5.65, 5.47, and 5.30. The differences between groups are statistically significant with a *p* < 0.05. Similarly, the mean score of the General Self-efficacy Scale and the Rosenberg Self-esteem Scale also show a higher value in the good mental health class than in the two remaining classes. These differences were also statistically significant (*p* < 0.05).

**TABLE 3:** Characteristics of participants according to three classes from the MHI-5 scale.

Characteristics	Poor mental health class		Fair mental health class		Good mental health class		Total		*p*-value
				
	*n*	%	*n*	%	*n*	%	*n*	%	
Total	51	14.33	166	46.63	139	39.04	356	100.00	
**Gender**									
Male	13	26.00	43	26.22	42	30.66	98	27.92	0.66
Female	37	74.00	121	73.78	95	69.34	253	72.08	
**Job**									
Students	45	88.24	146	87.95	128	92.09	319	89.61	0.47
Others	6	11.76	20	12.05	11	7.91	37	10.39	
**Education**									
High School or lower	42	82.35	137	82.53	117	84.17	296	83.15	0.92
Tertiary and upper	9	17.65	29	17.47	22	15.83	60	16.85	
**Marital**									
Single	50	98.04	157	94.58	130	93.53	337	94.66	0.47
Married	1	1.96	9	5.42	9	6.47	19	5.34	
**Household economy**									
Low	21	41.18	53	31.93	36	25.9	110	30.90	0.25
Average	19	37.25	66	39.76	55	39.57	140	39.33	
High	11	21.57	47	28.31	48	34.53	106	29.78	
**Acute symptoms**									
None	12	23.53	51	30.72	50	35.97	113	31.74	0.53
1	4	7.84	13	7.83	12	8.63	29	8.15	
> = 2	35	68.63	102	61.45	77	55.40	214	60.11	
**Chronic diseases**									
No	45	88.24	156	93.98	130	93.53	331	92.98	0.36
Yes	6	11.76	10	6.02	9	6.47	25	7.02	
**Binge drinking**									
No	37	72.55	126	76.83	110	79.14	273	77.12	0.63
Yes	14	27.45	38	23.17	29	20.86	81	22.88	
**Hazardous drinking**									
No	39	76.47	141	84.94	120	86.33	300	84.27	0.24
Yes	12	23.53	25	15.06	19	13.67	56	15.73	

	**Mean**	**SD**	**Mean**	**SD**	**Mean**	**SD**	**Mean**	**SD**	***p*** **-value**

Age	21.92	2.37	22.01	3.34	22.09	3.33	22.03	3.21	0.72
**Measurement of perceived social support (MSPSS)**									
Family (range 0 to 7)	4.42	1.18	5.16	0.96	5.65	0.98	5.24	1.08	<0.01
Friends (range 0 to 7)	4.59	0.93	4.96	0.87	5.47	0.77	5.10	0.90	<0.01
Significant others (range 0 to 7)	4.30	1.09	4.81	0.92	5.30	0.97	4.93	1.02	<0.01
General Self-efficacy scale (range: 0 to 40)	25.78	2.69	28.14	3.04	29.72	2.97	28.42	3.23	<0.01
Rosenberg Self-esteem scale (range: 0 to 30)	21.35	5.13	26.50	4.25	29.53	4.01	26.94	5.06	<0.01

### Selected results of multinomial logistic regression: Prediction of the MHI-5 patterns

In Table 4 compared to participants with a low household economy, those with an average household economy had 2.11 and 4.79 times higher odds of being in a good mental health class relative to those with a fair mental health class (OR = 2.11; 95%CI: 1.07; 4.14; *p* < 0.05) and a poor mental health class (OR = 4.79; 95%CI: 1.51; 15.20; *p* < 0.001).

**TABLE 4:** Selected results of multinomial logistic regression: Prediction of the patterns of the MHI-5.

Characteristics	Good vs. Fair mental health class		Fair vs. Poor mental health class		Good vs. Poor mental health class	
			
	OR	95%CI	OR	95%CI	OR	95%CI
**Gender** (Ref. Male)						
Female	0.67	0.36; 1.27	0.59	0.21; 1.65	0.40	0.13; 1.23
**Age** (unit: year)	1.09	0.96; 1.24	1.08	0.88; 1.32	1.18	0.94; 1.48
**Education** (Ref. High School or lower)						
Tertiary and upper	0.55	0.19; 1.55	0.60	0.12; 3.03	0.33	0.05; 1.99
**Marital status** (Ref. Single)						
Married	2.19	0.49; 9.83	2.43	0.14; 41.16	5.32	0.25; 114.15
**Job** (Ref. Students)						
Others	3.36*	0.88; 12.74	1.92	0.32; 11.68	6.46*	0.79; 53.12
**Household economy** (Ref. Low)						
Average	2.11**	1.07; 4.14	2.28	0.82; 6.33	4.79***	1.51; 15.20
High	1.91*	0.95; 3.85	1.32	0.46; 3.82	2.52	0.77; 8.29
**Acute symptoms** (Ref. 0)						
1	0.98	0.37; 2.60	0.47	0.07; 2.99	0.46	0.06; 3.30
≥ 2	0.63	0.35; 1.11	0.41*	0.16; 1.06	0.26**	0.09; 0.73
**Chronic diseases** (Ref. No)						
Yes	1.49	0.49; 4.52	0.55	0.12; 2.53	0.82	0.15; 4.59
**Binge drinking** (Ref. No)						
Yes	0.72	0.30; 1.70	1.00	0.24; 4.12	0.71	0.15; 3.40
**Hazardous drinking** (Ref. No)						
Yes	1.16	0.43; 3.14	0.66	0.15; 3.02	0.77	0.14; 4.18
**Measurement of perceived social support (MSPSS)**						
Family (unit: one score)	1.24	0.93; 1.65	1.80***	1.16; 2.80	2.23***	1.36; 3.64
Friends (unit: one score)	1.87***	1.24; 2.80	0.84	0.47; 1.50	1.57	0.81; 3.04
Significant others (unit: one score)	1.07	0.75; 1.53	1.31	0.77; 2.22	1.40	0.77; 2.52
**General Self-efficacy scale** (unit: one score)	1.05	0.94; 1.17	1.11	0.95; 1.31	1.17*	0.97; 1.40
**Rosenberg Self-esteem scale** (unit: one score)	1.17***	1.08; 1.26	1.26***	1.13; 1.41	1.47***	1.30; 1.67

***p < 0.01, **p < 0.05, *p < 0.1.

Furthermore, the odds ratio for participants with more than two acute symptoms was 0.26 (OR = 0.26, 95%CI: 0.09; 0.73), indicating that those had 3.85 times higher odds of being in a good mental health class relative to a poor mental health class, as compared to those without acute symptoms.

Regarding the measurement of the perceived social support scale, people who received higher support from their family had a higher odds ratio of being in fair and good mental health classes about 1.80 times (OR = 1.80; 95%CI = 1.16; 2.80) and 2.23 times (OR = 2.23; 95%CI = 1.36; 3.64) than in poor mental health class.

Similarly, participants who received the highest support from their friends were more likely in the good mental health class compared to the fair mental health class by 1.87 times (OR = 1.87; 95%CI = 1.24; 2.80).

Related to Rosenberg’s self-esteem scale, participants have 1.17, 1.26, and 1.47 times higher odds of being in a good compared to fair mental health class (OR = 1.17, 95%CI: 1.08; 1.26), fair compared to poor mental health class (OR = 1.26, 95%CI: 1.13; 1.41), and good compared to poor mental health class (OR = 1.47, 95%CI: 1.30; 1.67), as Rosenberg’s self-esteem score increased by 1 unit.

## Discussion

The current study was considered the first work in Vietnam using the LPA approach to explore unobserved subgroups of the mental health status of the young population. Our findings determined three latent subgroups based on the MHI-5 score from the study samples. The majority of respondents were classified as having fair mental health (46.63%), while those who were assessed as having poor mental health accounted for the smallest proportion (14,33%). Notably, receiving family and friend support (according to the MSPSS scale) was significantly related to having better mental health than the other groups. Moreover, it was the same case with the high individual’s self-esteem score as well as the household income aspect, which was at an average level compared to other groups. People with more than two acute symptoms, on the other hand, saw their mental health get worse in a big way.

In detecting mental health issues, the MHI-5 was proved to be the comprehensive and commonly used General Health Questionnaire (Goldberg and Blackwell, 1970; Berwick et al., 1991). This measurement has good performance characteristics in screening for mental health issues in the general population (Rumpf et al., 2001). Moreover, the MHI-5 was widely utilized as a mental component of the SF-36 scale in previous studies in Vietnam (Watkins et al., 2000). Therefore, we decided to use the MHI-5 as a screening tool to classify people through LPA analysis. The percentage of mental health symptoms in the present study was consistent with the UNICEF report in 2015, with the prevalence of general mental health problems in Vietnam ranging from 8 to 29% (Unicef Viet Nam, 2020). In 2019, similar data was also identified with 14.4% of common mental disorders through a population-based cross-sectional survey (Nguyen T. et al., 2019). According to the Vietnam National Mental Hospital, the prevalence of 10 common disorders occupied 14.2% in 2014 (World Health Organization, 2021). Compared to a latent profile study using the MHI-5 scale for screening mental health status (with three latent profiles from poor to good mental health) among Australian women (2015), that identified people who were still alive between 1996 and 2008 had poor mental health were 28 and 17.4%, respectively (Leigh et al., 2016). Furthermore, our respondents were classified into fair and good mental health, those who perceived social support at high levels from family and friends, compared to participants identified with poor mental health status. Indeed, community support was of the highest importance when an individual suffered health issues. This point was demonstrated in the previous research (Bloor et al., 2004; Reblin and Uchino, 2008; Uchino et al., 2012). In addition, many scholars showed evidence that social supports such as family and significant others have effects on health issues, for example, the neuroendocrine, immune functions (Uchino, 2006), and cardiovascular responses (Uno et al., 2002). They also argued that the effectiveness of social support brought a buffering effect on depression and stressful life events (Komatsu et al., 2010), leading to a positive effect on mental health (Veselska et al., 2010). Our results emphasized that family and significant others seemed to make the greatest contribution to good mental health status and chronic disease (Vassilev et al., 2013).

Likewise, people with higher Rosenberg self-esteem scores are more likely to be in the better mental health groups, according to the current findings. In 2001, Richard et al. indicated regulation of emotional states was affected mainly by self-esteem (Robins et al., 2001). People with high self-esteem could regulate their emotional states by reducing negative thoughts and concerns about potential threats (Brown and Marshall, 2001). According to Watson et al. (1988), low self-esteem individuals tend to have a negative self view, whereas high termed positive self-esteem individuals tend to be secure and satisfied with themselves (Watson et al., 1988). Various researchers also found a correlation between low mental health and general self-esteem (Henriksen et al., 2017; Nguyen D. T. et al., 2019). Many studies had declared that a good mood reduces self-control because a happy (vs. unhappy) status tended to prolong the time for positive mood & less mental illness symptoms (Isen and Simmonds, 1978; Wegener and Petty, 1994). Moreover, participants with an average income compared to those with a low-income were more likely to be in better mental health groups. This result was completely consistent with a population-based cross-sectional survey in Vietnam’s southern provinces, which showed participants who had mid-level or advantaged economic status were less likely to have family members with any psychotic symptoms (Nguyen T. et al., 2019). At the individual level, psychotic symptoms may onset coinciding with adverse events which are influenced by factors at the household level such as financial constraints or lack of social support (Dutta et al., 2019). Negative effects of economic shocks such as financial crises, getting fired, or income loss according to the household aspect of mental health status, which was demonstrated by previous studies (Baird et al., 2013; Marcus, 2013; Adhvaryu et al., 2019).

One of the notable results was that participants who experienced more than two acute symptoms had a significant association with deteriorating mental health. Previous studies indicated the inverse association between acute diseases and mental illnesses such as stress, anxiety, depression, etc. These issues often occur when people are hospitalized with severe illnesses. The health status of those with more mental illness tended to deteriorate during and after hospitalization (Covinsky et al., 1997). The systematic review research result was implemented among survivors of cancer in 134 publications and indicated similarity in the above-discussed point with the effects of acute symptoms of cancer diseases and mental health status on survivorship (Van Leeuwen et al., 2018). Furthermore, in Switzerland, a retrospective study identified that a quarter of patients who frequently used the emergency department suffered from mental disorders, and those were more likely to be in personality disorders (Slankamenac et al., 2020). Recently, numerous epidemic outbreaks led to mental health changes followed by an increase in confirmed cases, such as the COVID-19 pandemic, Middle East Respiratory Syndrome, etc., (Jeong et al., 2016; Shi et al., 2020). These contexts could lead to depressive symptoms, anxiety, insomnia, acute stress, and latent risks among the general population.

Our findings demonstrated several implications that could be helpful to improve mental health status based on the discussed evidence. First and foremost, our results reflected the MHI-5 score, which corresponded to the middle and poor mental health classes among the study population. Thus, raising optimal mental health as well as health-related quality of life was presented with the warning need to further study and expand mental healthcare aspects. Second, the risk factors lead to mental illness prevention needed to address (1) general community education related to mental problems and prevention; (2) improved approaches and care for the high-risk groups; and (3) emphasis on limiting access to high-risk factors causing mental illness from the analyzed study, such as acute diseases, economic problems, and lack of social support. Third, we further posited that approaching mental problems based on the LPA analysis would explore the following implications: (1) social-supports played an important role (particularly with family & significant others) in mental health, thus, it was necessary to increase an intimate connection between individuals, families, and communities; (2) people with higher Rosenberg self-esteem scores were found to have better mental health status, so future intervention strategies may refer to our findings for comprehensive effectiveness; and (3) policies development to promote and improve the mental health in the general community and strengthen the health care system.

This study provided a valuable picture of the mental health of the general population and specifically young people in Vietnam. However, the findings of our study also entailed several limitations and sources of bias. First, no causal conclusions could be drawn because our research used a cross-sectional study. Second, a lack of participation on the part of those respondents who were identified as having mental problems was probably caused by selection bias. This would be challenging to prevent because participants attended voluntarily and there was no effort to access the mental patients. Third, the majority of participants were female (72.1%) and students (89.6%), so the study sample was non-representative of urban young adults in Vietnam. Moreover, we were not able to get a larger and more complete sample due to participants recruited during the outbreak of COVID-19. Hence, future research needs to extend the sample collection and ensure the representation of the study population. Hence, future research is needed to extend the sample collection. For future research, it would be helpful to consider the social-ecological effects of using the MHI-5 scale to evaluate the general Vietnamese population, especially the loneliness scores, which could be used to help formulate policies to improve mental health in the community.

## Conclusion

We found substantial evidence that the variables including self-esteem, perceived social support, and household economy on average are associated with higher profiles of mental health status. The impacts of acute symptoms on mental health outcomes were still insufficient, but our findings showed evidence that greater acute symptoms are related to more mental problems. For this study, evidence was given to raise awareness of mental health in the community and develop policies to promote social support and strengthen the general health care system.

## Data availability statement

The raw data supporting the conclusions of this article will be made available by the authors, without undue reservation.

## Ethics statement

The studies involving human participants were reviewed and approved by the Institutional Ethical Review Board in Hanoi Medical University, Vietnam. The patients/participants provided their written informed consent to participate in this study.

## Author contributions

TTPN, TV, HD, GV, CL, BH, RH, and CH: conceptualization. TTPN, TTN, VD, and AT: data curation. TTPN, TTN, and VD: formal analysis. TTPN, TV, HD, CL, BH, RH, and CH: methodology. TTPN, TV, HD, GV, and AT: supervision. TTN and VD: investigation. TTPN, TTN, VD, and GV: writing – original draft. TTPN, TTN, VD, TV, HD, GV, AT, CL, BH, RH, and CH: writing – review and editing. All authors contributed to the article and approved the submitted version.
